# The Practice of Surgery

**Published:** 1898-09

**Authors:** 


**The Practice of Surgery.** By HENRY R. WHARTON, M.D., and B. FARQUHAR CURTIS, M.D. Pp. vii., 1240. Philadelphia: J. B. Lippincott Company. 1898.

We have much to say in favour of this work, which attempts the condensation of the practice of surgery within a single volume. With few exceptions, the text is quite modern, particularly from the point of view of treatment; the majority of the illustrations are good, and the authors are to be congratulated on a difficult task. There are some parts, however, the perusal of which seems unsatisfactory, as they lack the definition and finish of many standard English text-books. We may refer for instance to the chapter on septicæmia (which makes a distinction between sapræmia and septic "intoxication"), to diffuse lipoma, to the symptoms of glanders, to the absence of a good description of myelitis, of complications of mastoid disease, and of the surgery of the breast—one page only being allotted to cysts of the breast. The statement that in erysipelas the lymph glands are not generally infected, although they become so if cellulitis is present, is not in accordance with our experience. The chapter on the conditions affecting the results of operation is an excellent one. The text throughout is freely elucidated by illustrations of various kinds; we must, however, take exception to those which represent the reprehensible practice of bandaging a limb beneath a splint.

